# Preferences and attitudes of physicians in India towards continuing medical education

**DOI:** 10.1080/21614083.2017.1332940

**Published:** 2017-06-19

**Authors:** Manan D. Shah, Vishal Goyal, Vikram Singh, Jayesh Lele

**Affiliations:** ^a^ Medical Affairs, Janssen India, Johnson & Johnson Pvt. Ltd, Mumbai, India; ^b^ Indian Medical Association, Mumbai, Maharashtra, India

**Keywords:** Continuing medical education, physicians, questionnaire, survey

## Abstract

**Introduction**: Physicians in India display an enthusiasm for continuing medical education (CME), however a proper system for facilitation and organisation of CME activities is yet to evolve in the country.

**Method**s: A questionnaire-based survey was conducted among 751 physicians from eight medical specialties across India and the data retrieved was analysed at individual physician and collective specialty-specific levels.

**Results**: The surveyed physicians considered case presentations (73%), live speaker programmes (70%) and round-table meetings/focus group meetings (70%) as the most effective CME activities. They preferred a duration of <2 h for CME activities such as webcasts (89%) and lectures (86%). Most of them considered scientific agenda (78%) and stature of speakers (77%) as the most important determinants of the quality of a CME event. Most physicians wanted topics such as disease guidelines (88%) and new drugs/devices/interventions (86%) for discussion in CME activities. Medical associations (87%) were the most desirable organisations for holding the CME activities and face-to-face modules appealed to majority of the physicians (64%).

**Conclusion**s: This study indicates that Indian physicians prefer live, interactive, short, specialty-specific educational sessions for CME activities, delivered by Indian experts and organised by medical associations at domestic destinations.

## Introduction

Continuing medical education (CME) is defined as the educational activities that serve to maintain, develop or increase the knowledge, skills, performance and relationships that a medical practitioner uses to provide services to patients, the public or the profession [1]. Some of the state medical councils in India, such as those of Maharashtra and Gujarat states, mandate a certain number of CME training hours for each physician during a specified period [2,3].

There are knowledge gaps pertaining to the format, technology and logistics behind the organisation of a CME event. CME strategies need to be honed to meet the needs of their target audience: the physicians. The preferences and requirements of the different medical specialties may also have to be incorporated into the design of the CME programmes. Hence, it is necessary to elucidate the diverse factors that form the foundation of a successful CME event.

The objective of our survey was to understand the preferences of Indian physicians with respect to CME activities and the compliance issues that they face in this regard. The study sought to understand the physicians’ choices of CME format and media. The study observed the attitudes and behaviours of physicians that influenced the effectiveness of CME activities. The study also explored the benefits and outcomes of CME events conducted for physicians from various specialties across India.

## Research design and methods

### Participants

A questionnaire (Supplementary Document 1) was prepared and validated using a face-to-face interview with 55 selected physicians. The physicians’ list was provided by Janssen across specialties such as medical oncology, psychiatry, diabetology and endocrinology, rheumatology, dermatology, orthopaedics, neurology and general practitioners/family physicians. The physicians were selected randomly after stratification of geography and age. The questionnaire comprised queries focusing on demographics, medical specialty and CME activities. A questionnaire-based survey on paper was conducted among 751 Indian physicians affiliated to medical oncology, psychiatry, diabetology (including endocrinology), rheumatology, dermatology, orthopaedics, neurology, general practitioners and family physicians. The physicians were instructed to select from multiple options and/or rate their preferred options on a scale of 1 to 5 (1: least effective; 2: not effective; 3: neutral; 4: effective; 5: most effective).

### Statistical analysis

Data were summarised with descriptive statistics, mean and standard deviation [SD] for continuous variables, and frequency and percentages for categorical variables. Analysis was performed on collective as well as specialty-specific data. Analysis of collective data is presented in the main text and tables, while data regarding each specialty are presented in the supplementary tables.

## Results

Most of the participating physicians were males (86%), who belonged to the age group 41–50 years (40%). Clinical experience exceeding 10 years was common (72%). Sixty four per cent of the physicians were self-employed and 78% of them were involved in private practice. The specialties most commonly represented in our survey were dermatology (23%) and orthopaedics (19%) (Table 1).

**Table 1.** Participants’ demographics (*n* = 751).

Characteristics	
Age group (years)	
<40	26%
41–50	40%
51–60	24%
>60	9%
Gender	
Male	86%
Female	14%
City	
Metropolitan	65%
Smaller cities	35%
Specialty	
Medical oncology	11%
Psychiatry	7%
Diabetology	18%
Rheumatology	7%
Dermatology	23%
Orthopaedics	19%
Neurology	9%
General practitioners (and family physicians)	6%
Total years of practice	
1–5	9%
6–10	19%
11–15	23%
16–20	17%
>20	32%
Mean time spent on CME activities	3.35 h
Practice type	
Private	78%
Government	22%
Practice type	
Self-employed	64%
Employed	36%
Practice type	
Teaching institute	49%
Non-teaching institute	51%

CME, continuing medical education.

**Figure 1.**
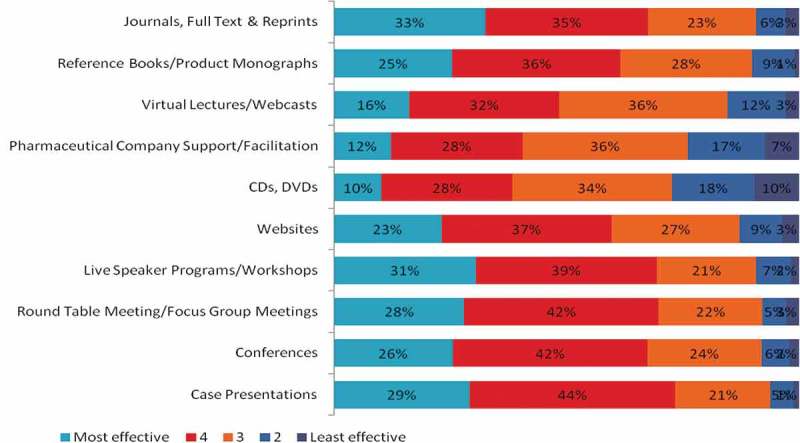
Effectiveness of different continuing medical education activities.

Most physicians preferred CME events that offered live and interactive educational sessions. Self-learning modules were preferred over instructor-led modules, and face-to-face modules were preferred over digital modules (Table 2 and Supplementary Table 1). Case presentations (73%), speaker programmes or workshops (70%), round-table meetings or focus group meetings (70%) and conferences (68%) were the commonly sought-after CME activities (Figure 1).

**Table 2.** Preferred aspects of continuing medical education (CME) activities.

Self-learning vs instructor led (*n* = 703)
Self-learning 59%
Instructor led 41%
Digital vs face to face (*n* = 709)
Digital 36%
Face to face 64%
Print vs digital (*n* = 698)
Print 44%
Digital 56%
Domestic location vs international location (*n* = 684)
Domestic location 75%
International location 25%
Indian speaker vs foreign speaker (*n* = 683)
Indian speaker 66%
Foreign speaker 34%
Residential vs non-residential (*n* = 673)
Residential 60%
Non-residential 40%
Credit points vs institute of repute (*n* = 667)
Credit points 53%
Institute of repute 47%

Nearly 60% of dermatologists and orthopaedicians acknowledged the effectiveness of reference books or product monographs for CME, but they considered CDs or DVDs to be less effective. Case presentation was considered an effective format by the majority of diabetologists and oncologists. The use of internet or virtual media for CME also garnered the attention of many physicians (Supplementary Table 2).

**Figure 2.**
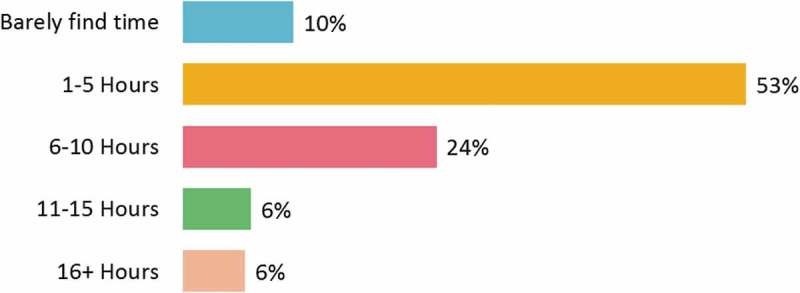
Time spent on the internet (online) for medical education purposes per week (*n* = 746).

**Figure 3.**
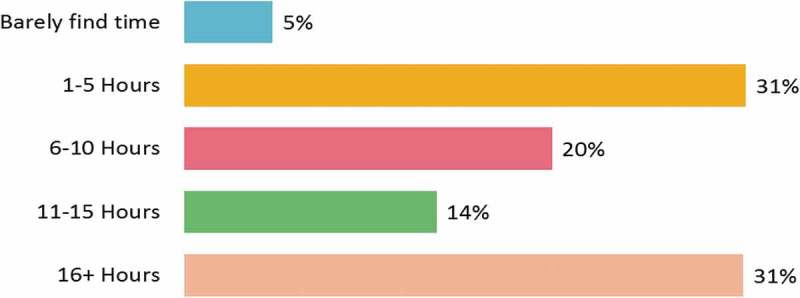
Time spent on all types of medical education activities per month (*n* = 746).

The physicians’ awareness of and requirement for CME were evident from the time they spent on the internet for CME-related activities. Overall, 77% of the physicians spent up to 10 h per week on the internet (Figure 2). However, the mean time spent by physicians on all CME activities was 3.35 h per month (Table 1 and Supplementary Table 3(a)). About half of the physicians were preoccupied with various CME activities for <10 h every month (Figure 3). Overall, 55% of the physicians were satisfied with the time they spent on the CME activity. General practitioners (68%) and diabetologists (63%) constituted the highest proportion of physicians who were satisfied with their CME participation, yet they reported little time spent on the internet for the same (Supplementary Tables 3(b,c)).

**Figure 4.**
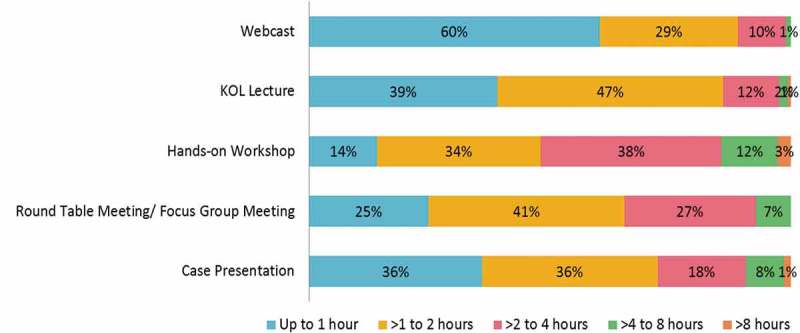
Duration of continuing medical education activities preferred by physicians (*n* = 743). KOL, key opinion leader.

**Figure 5.**
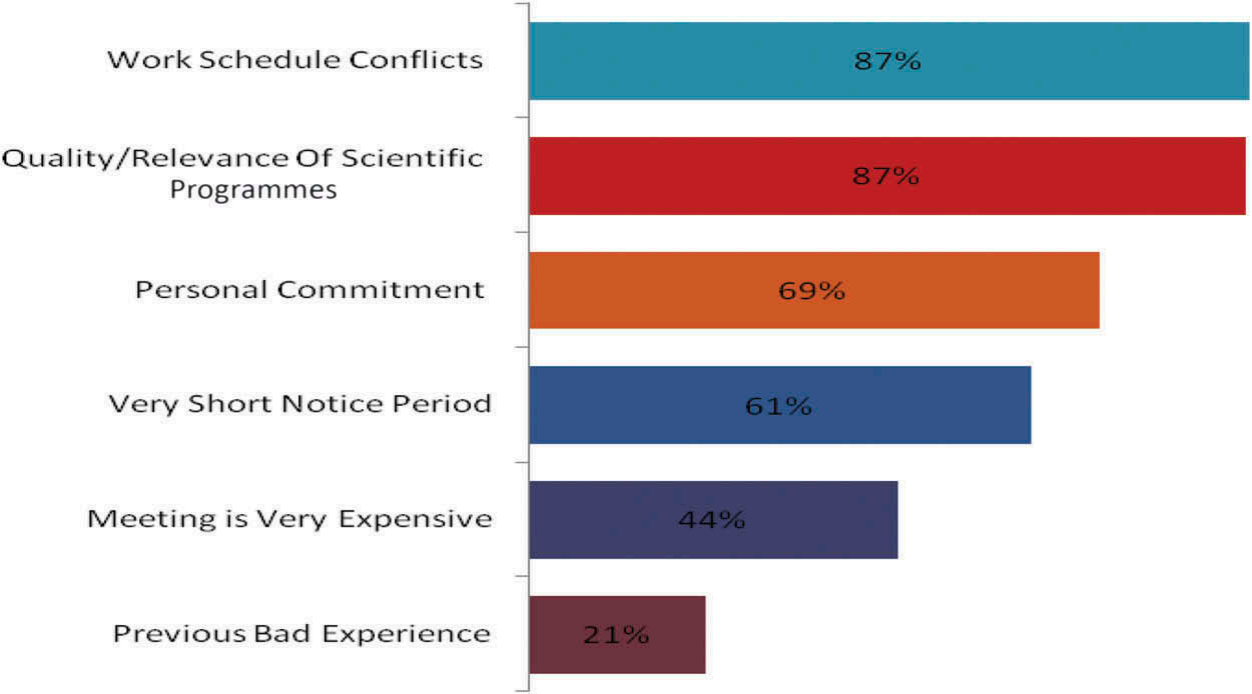
Factors responsible for non-attendance in a continuing medical education activity (*n* = 745).

The physicians also expected to spend only a limited amount of time on the live CME events. Most physicians preferred a duration of ≤2 h for webcasts, lectures, case presentations and round-table/focus group meetings (Figure 4). However, hands-on workshops of >2 h duration were preferred by the majority of dermatologists, oncologists and neurologists (Supplementary Table 4). Although the physicians received invitations to various CME events, they were able to attend only a few of them. Work schedule conflicts and quality of educational programmes were most often cited as the reasons for not attending a CME activity (Figure 5 and Supplementary Table 5(a)).

**Figure 6.**
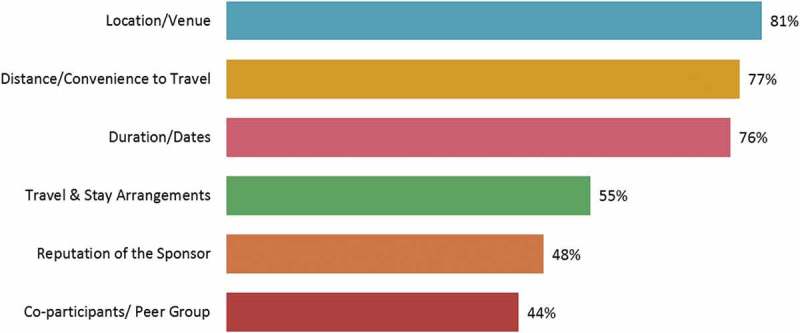
Most important aspect of continuing medical education activity.

The logistics of a CME event also influenced physician attendance. The three most important aspects affecting the decision to attend a CME activity were location or venue, duration or dates of the event, and the distance to travel or convenience of travel (Figure 6 and Supplementary Table 5(b)). Seventy five per cent of the physicians preferred a domestic location for CME events, and 66% of them preferred Indian speakers over foreign experts. Residential CME activities were preferred by 60% of the physicians (Table 2).

**Figure 7.**
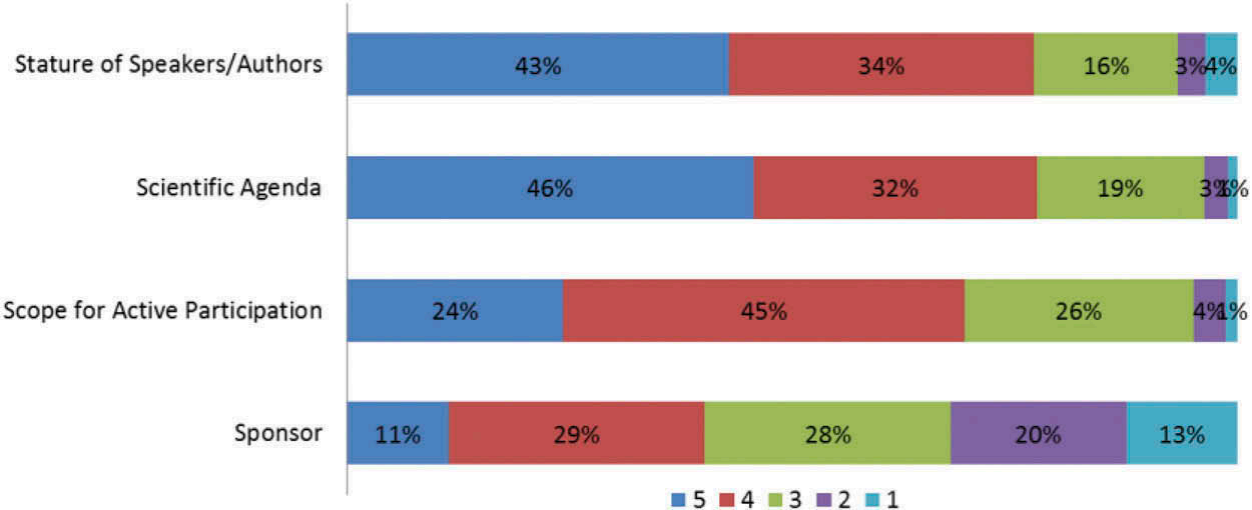
Preferred characteristics of continuing medical education activities on a scale of 1–5 (1: least preferred; 5: most preferred).

A physician’s decision to attend a CME event was influenced by the attributes of the CME programme. Most physicians considered the scientific agenda, stature of the speakers and scope for active participation as the most important determinants of the quality of a CME event (Figure 7 and Supplementary Table 6). Most physicians also considered the reputation of the host institute as a more significant criterion than the allocated credit hours (Table 2 and Supplementary Table 1).

**Figure 8.**
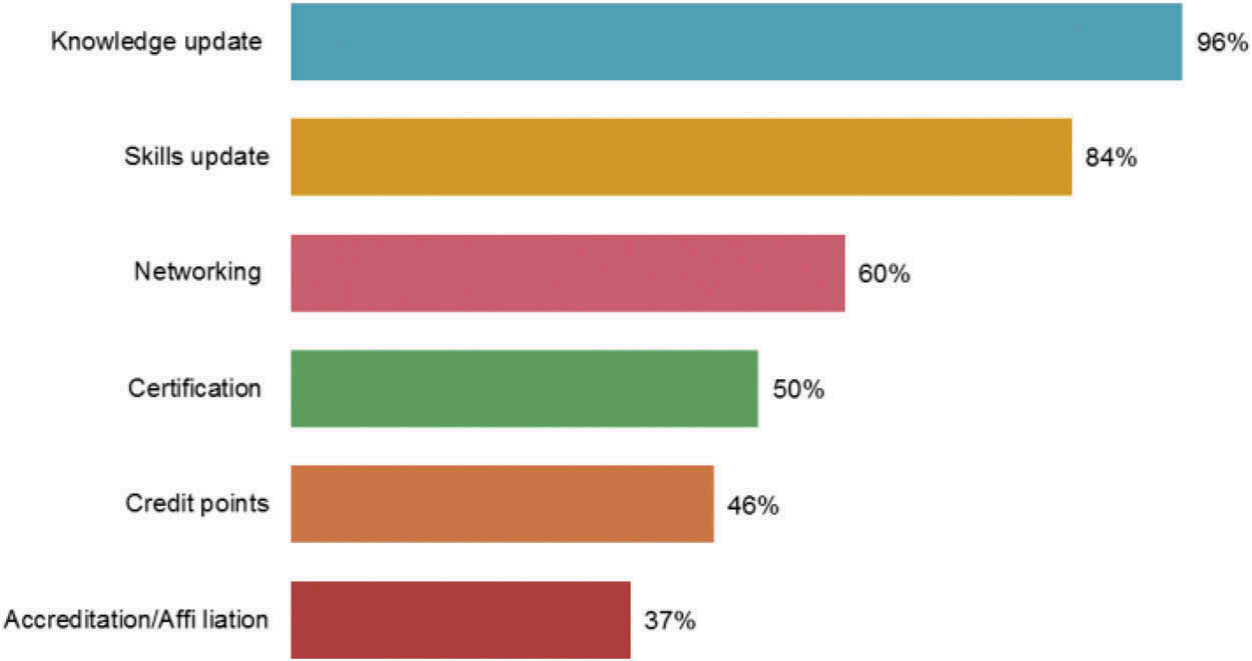
Desired benefits/outcomes of continuing medical education activity.

Most physicians attended the CME events because they wanted to update their knowledge and skills through the activities (Figure 8). There was no statistically significant difference in terms of the knowledge or skills update between the West zone and the other zones of the country. Furthermore, the proportion of candidates interested in networking, certification and credit points was found to be significantly less in the West zone compared to the rest of the country. Similarly, the proportion of candidates interested in accreditation/affiliation was found to be significantly less in the West zone compared to the rest of the country.

**Figure 9.**
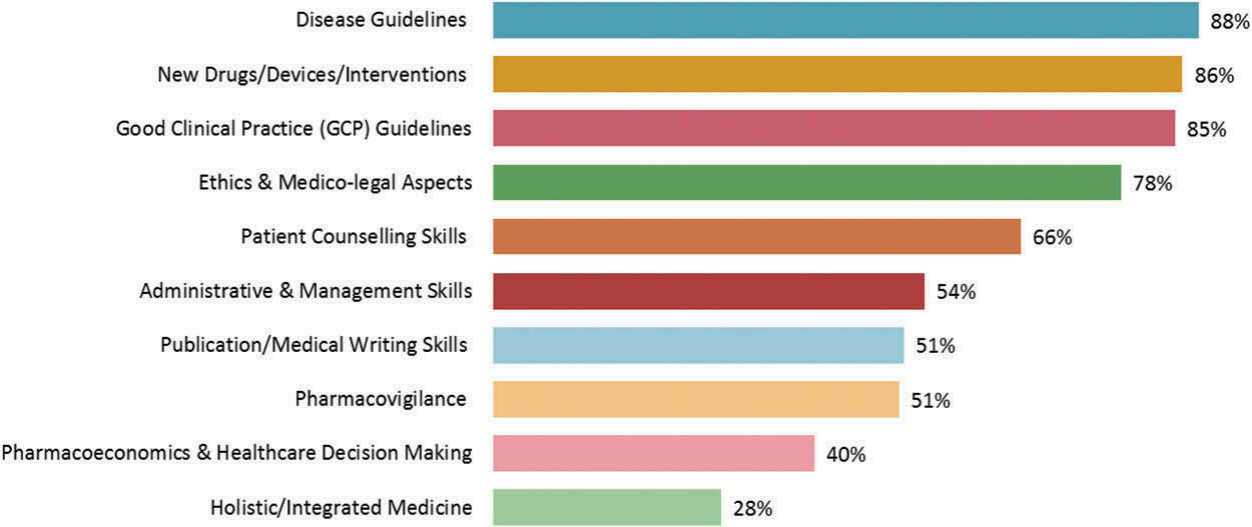
Subject preferred by physicians for discussion at continuing medical education events (*n* = 744).

The general discussion topics preferred by the physicians included disease guidelines, new drugs/devices/interventions and the clinical practice guidelines (Figure 9). However, the specific clinical topics for discussion at CME events varied according to the specialty of the physician. Seventy-two per cent of orthopaedicians were keen to develop administrative and management skills, while only 33% of them were interested in pharmacoeconomics and healthcare decision making (Supplementary Table 7).

**Figure 10.**
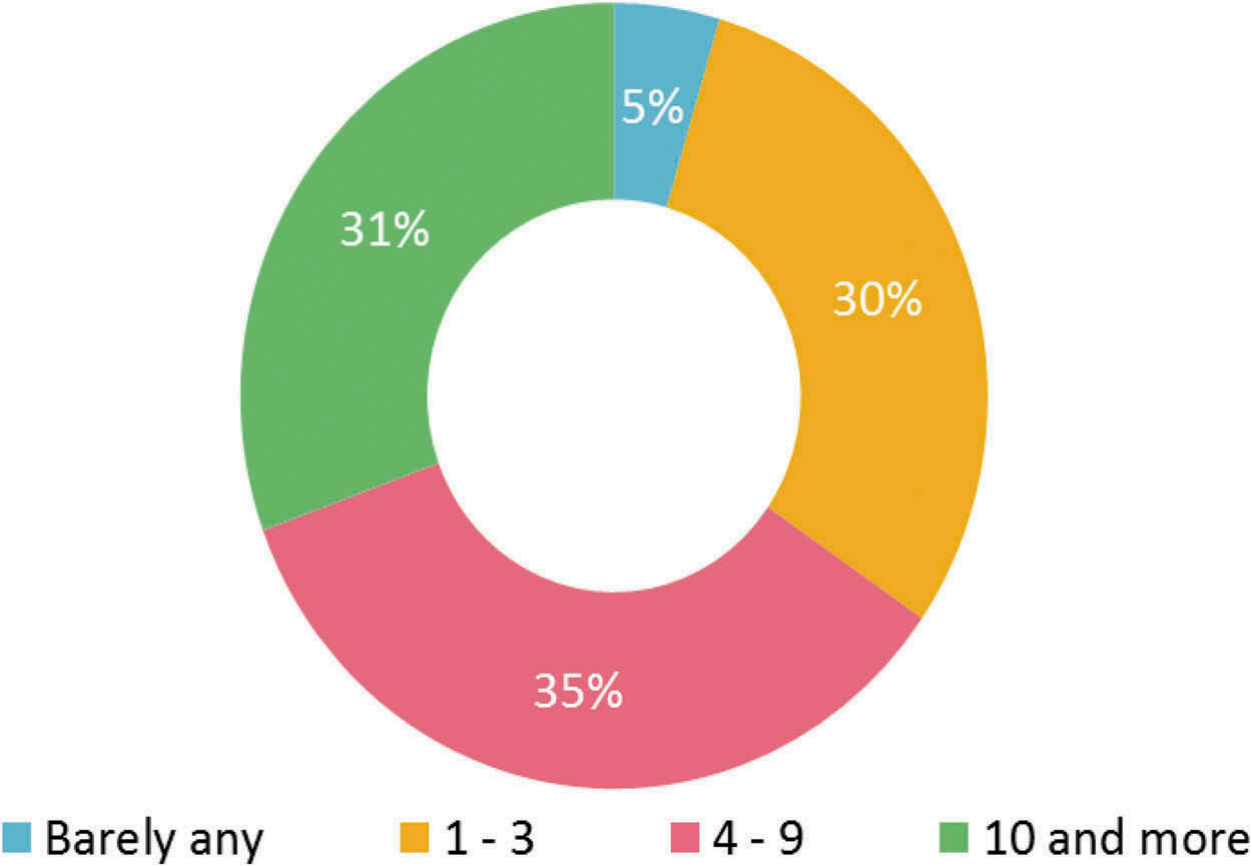
Invitations received to attend certified continued medical education courses per year (*n* = 723).

In addition to organising CME events that appealed to the interests of the physicians, the organisers actively pursued them by sending invitations. Approximately two-thirds of the physicians received more than three invitations per year to attend accredited CME courses (Figure 10). A greater proportion of oncologists (75%) and dermatologists (73%) received more than three invitations compared to physicians of other specialties (Supplementary Table 8).

**Figure 11.**
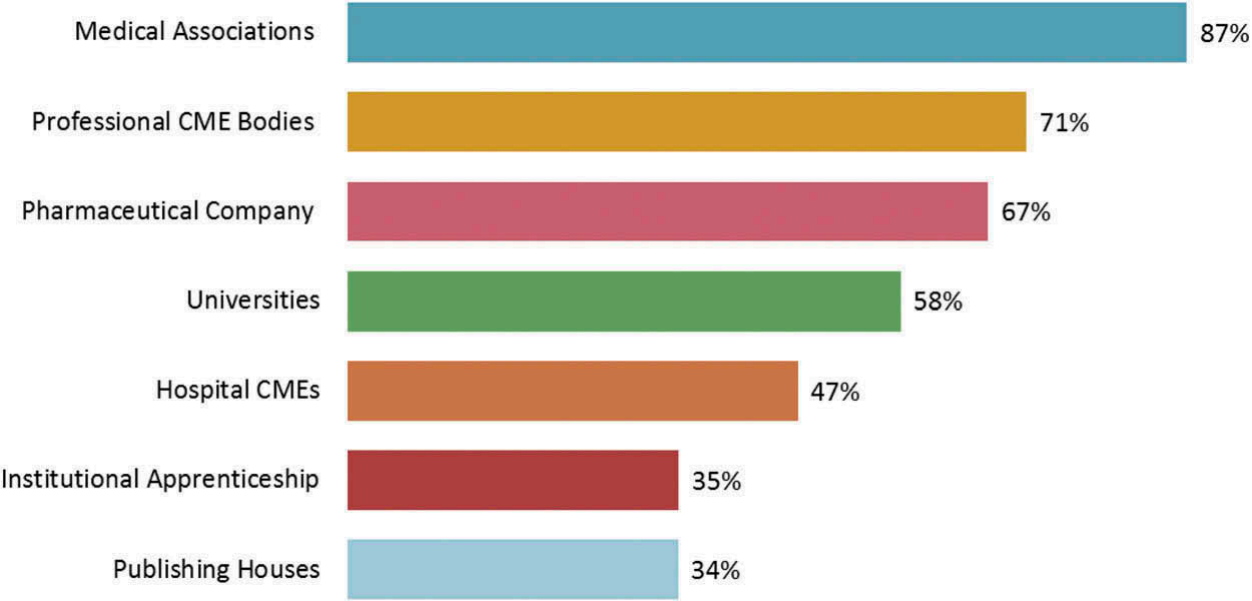
Organisations preferred by physicians for continuing medical education (CME) activities.

Medical associations were the most preferred CME organisations, followed by professional CME bodies and pharmaceutical companies (Figure 11). However, more invitations were received for non-accredited CME events than for certified CME events. Our findings suggest that physicians are well aware of accredited and non-accredited CME events, and attend them based on their merit.

## Discussion

Assessment of the unmet needs of physicians in India is a sound approach for evaluating the *status quo* of CME activities. In this cross-sectional study, 751 physicians from various specialties across India responded to a questionnaire focusing on CME programmes. The survey queried the physicians about the format, technology and logistics that make a CME event successful and desirable.

Our study suggests that Indian physicians prefer live, interactive CME activities. The majority of physicians in our study had more than 10 years of clinical experience and most of them (64%) spent more than 5 h per month on CME activities. The physicians affiliated to different specialties actively participated in related online activities. The physicians were aware of the accreditation status of the CME activities and preferred attending specialty-specific CME activities to obtain credit hours in compliance with the mandatory policies of the state medical councils. However, there was no significant difference in physicians’ preferences and attitudes in the West zone, where state medical councils have CME requirements and mandatory policies to comply with (e.g. in Gujarat and Maharashtra), compared with other zones included in the survey.

The physicians preferred CME events organised at domestic locations and hosted by Indian speakers of repute. Overall, the physicians were interested in updating their knowledge on diseases, good clinical practice guidelines and novel therapies through official medical associations and professional CME bodies. The systematic review by Marinopoulos *et al*. confirmed that CME was effective in enabling the physicians to improve their knowledge, attitudes and practices [4]. Hence, there is enormous scope for the development and evolution of CME programmes orientated towards the physicians of India.

Our survey indicated that Indian physicians preferred CME activities of an interpersonal nature such as case presentations, live speaker programmes, round-table/focus group meetings, and conferences that focused on knowledge and skill enhancement. This is consistent with the findings of the study by Saha *et al*., who conducted a survey of diverse participants from a health conference. The study found that live events such as lectures were preferred by the majority of participants [5].

Our study also indicated that most physicians preferred specialty-specific CME events comprising topics that update them on areas related to their area of work. This is consistent with the findings of the study by Bjerre *et al*. on the content of CME events for Canadian physicians. This study identified the gaps in CME offerings by comparing the questions asked by physicians at the point of care with the content of CME courses [6].

Our findings suggest that CME events are being organised across India, and the organisers are spreading awareness about these programmes among physicians. As a result, the physicians are also keen to attend these events. The location, timing and content of CME events are important logistic factors. However, the scientific agenda and opportunities to actively participate in a CME programme were among the most important qualitative aspects of a CME programme.

One limitation of the survey is that the study population comprised predominantly males as it was conducted among specialty physicians. However, this is in line with well-known reports of women being substantially under-represented among specialty residencies.

Our study collected data from a large group of physicians affiliated to diverse specialties in India in order to understand their learning needs, knowledge gaps and preferred CME formats. Analysis of the data revealed that physicians in India prefer live, short, interactive, specialty-specific, focused, digitally enabled CME activities organised by medical associations and delivered by Indian experts. The results of this study are expected to aid the development of comprehensive, effective, scientific evidence-based CME activities.

## Lessons for practice

CME events conducted in India should include eminent Indian experts from reputed institutes; and the organised activities should also offer an opportunity for interaction with these experts. Indian physicians prefer learning about topics such as disease guidelines and novel therapies through short CME activities. Accredited CME activities may be available to physicians through CME events held in collaboration with medical associations or other recognised accreditation bodies such as university programmes. CME activities should be tailored to impart medical education that is relevant to the physicians’ specialty. The ultimate goal of a CME programme is to provide high-quality, unbiased medical educational activities that are based on the best evidence available to help the physicians to improve the patient outcomes within their scope of practice.

## Supplementary Material

CMEsurvey_supplementary_Tables.pptx

## References

[1] Prakash SS, Kavita GU, Shashikala P. Continuing medical education programs: earn or learn? J Edu Res Med Teach. 2014;2:1–7.

[2] New policy for holding the CM Es & Credit Points, Maharashtra Medical Council, Mumbai; p. 12 Available from: http://www.maharashtramedicalcouncil.in/CME/CMENotice/New%20CME%20Guidelines.pdf

[3] Gujarat Medical Council CME Guideline; Available from: http://www.gmcgujarat.org/cme_guidenline.aspx

[4] Marinopoulos SS, Dorman T, Ratanawongsa N, et al. Effectiveness of continuing medical education. Evidence Report/Technology Assessment No. 149 (Prepared by the Johns Hopkins Evidence-based Practice Center, under Contract No. 290-02-0018.) In: AHRQ Publication No. 07-E006 Rockville (MD): Agency for Healthcare Research and Quality; 2007.

[5] Saha A, Poddar E, Mankad M. Effectiveness of different methods of health education: a comparative assessment in a scientific conference. BMC Public Health. 2005;5:88. 16111502 10.1186/1471-2458-5-88 PMC1199606

[6] Bjerre LM, Paterson NR, McGowan J, et al Do continuing medical education (CME) events cover the content physicians want to know? A content analysis of CME offerings. J Contin Educ Health Prof. 2015;35:27–37. 25799970 10.1002/chp.21268

