# Supplementary material for: Preferences and attitudes of physicians in India towards continuing medical education
Source: J Eur CME. 2017 Jun 19;6(1):1332940. doi: 10.1080/21614083.2017.1332940 (PMC5843049; doi:10.1080/21614083.2017.1332940)
Supplement: CMEsurvey_supplementary_Tables.pptx [file ZJEC_A_1332940_SM6948.pptx]

## Slide 1
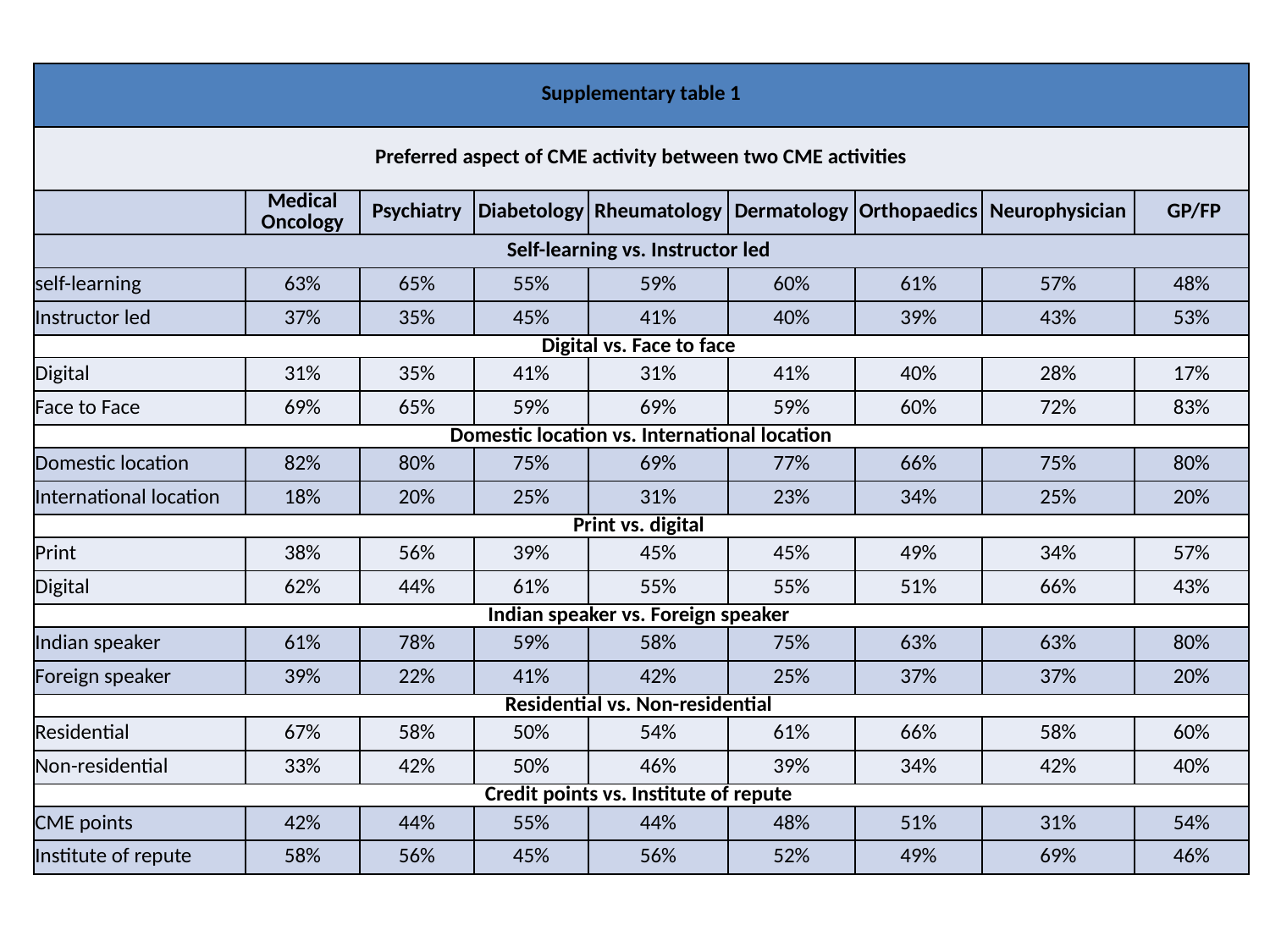

| Supplementary table 1 | | | | | | | | |
| --- | --- | --- | --- | --- | --- | --- | --- | --- |
| Preferred aspect of CME activity between two CME activities | | | | | | | | |
| | Medical Oncology | Psychiatry | Diabetology | Rheumatology | Dermatology | Orthopaedics | Neurophysician | GP/FP |
| Self-learning vs. Instructor led | | | | | | | | |
| self-learning | 63% | 65% | 55% | 59% | 60% | 61% | 57% | 48% |
| Instructor led | 37% | 35% | 45% | 41% | 40% | 39% | 43% | 53% |
| Digital vs. Face to face | | | | | | | | |
| Digital | 31% | 35% | 41% | 31% | 41% | 40% | 28% | 17% |
| Face to Face | 69% | 65% | 59% | 69% | 59% | 60% | 72% | 83% |
| Domestic location vs. International location | | | | | | | | |
| Domestic location | 82% | 80% | 75% | 69% | 77% | 66% | 75% | 80% |
| International location | 18% | 20% | 25% | 31% | 23% | 34% | 25% | 20% |
| Print vs. digital | | | | | | | | |
| Print | 38% | 56% | 39% | 45% | 45% | 49% | 34% | 57% |
| Digital | 62% | 44% | 61% | 55% | 55% | 51% | 66% | 43% |
| Indian speaker vs. Foreign speaker | | | | | | | | |
| Indian speaker | 61% | 78% | 59% | 58% | 75% | 63% | 63% | 80% |
| Foreign speaker | 39% | 22% | 41% | 42% | 25% | 37% | 37% | 20% |
| Residential vs. Non-residential | | | | | | | | |
| Residential | 67% | 58% | 50% | 54% | 61% | 66% | 58% | 60% |
| Non-residential | 33% | 42% | 50% | 46% | 39% | 34% | 42% | 40% |
| Credit points vs. Institute of repute | | | | | | | | |
| CME points | 42% | 44% | 55% | 44% | 48% | 51% | 31% | 54% |
| Institute of repute | 58% | 56% | 45% | 56% | 52% | 49% | 69% | 46% |

## Slide 2
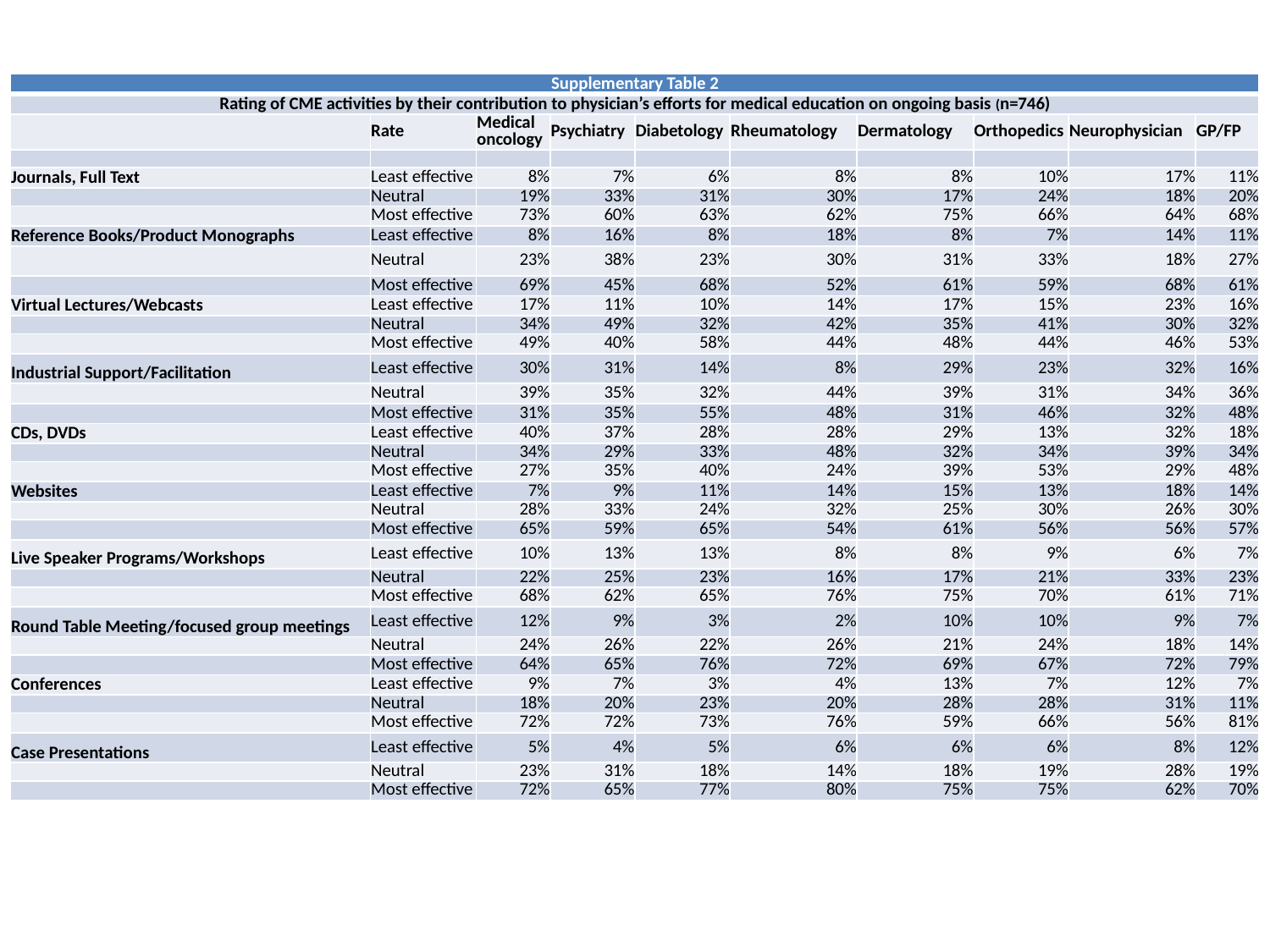

| Supplementary Table 2 | | | | | | | | | |
| --- | --- | --- | --- | --- | --- | --- | --- | --- | --- |
| Rating of CME activities by their contribution to physician’s efforts for medical education on ongoing basis (n=746) | | | | | | | | | |
| | Rate | Medical oncology | Psychiatry | Diabetology | Rheumatology | Dermatology | Orthopedics | Neurophysician | GP/FP |
| | | | | | | | | | |
| Journals, Full Text | Least effective | 8% | 7% | 6% | 8% | 8% | 10% | 17% | 11% |
| | Neutral | 19% | 33% | 31% | 30% | 17% | 24% | 18% | 20% |
| | Most effective | 73% | 60% | 63% | 62% | 75% | 66% | 64% | 68% |
| Reference Books/Product Monographs | Least effective | 8% | 16% | 8% | 18% | 8% | 7% | 14% | 11% |
| | Neutral | 23% | 38% | 23% | 30% | 31% | 33% | 18% | 27% |
| | Most effective | 69% | 45% | 68% | 52% | 61% | 59% | 68% | 61% |
| Virtual Lectures/Webcasts | Least effective | 17% | 11% | 10% | 14% | 17% | 15% | 23% | 16% |
| | Neutral | 34% | 49% | 32% | 42% | 35% | 41% | 30% | 32% |
| | Most effective | 49% | 40% | 58% | 44% | 48% | 44% | 46% | 53% |
| Industrial Support/Facilitation | Least effective | 30% | 31% | 14% | 8% | 29% | 23% | 32% | 16% |
| | Neutral | 39% | 35% | 32% | 44% | 39% | 31% | 34% | 36% |
| | Most effective | 31% | 35% | 55% | 48% | 31% | 46% | 32% | 48% |
| CDs, DVDs | Least effective | 40% | 37% | 28% | 28% | 29% | 13% | 32% | 18% |
| | Neutral | 34% | 29% | 33% | 48% | 32% | 34% | 39% | 34% |
| | Most effective | 27% | 35% | 40% | 24% | 39% | 53% | 29% | 48% |
| Websites | Least effective | 7% | 9% | 11% | 14% | 15% | 13% | 18% | 14% |
| | Neutral | 28% | 33% | 24% | 32% | 25% | 30% | 26% | 30% |
| | Most effective | 65% | 59% | 65% | 54% | 61% | 56% | 56% | 57% |
| Live Speaker Programs/Workshops | Least effective | 10% | 13% | 13% | 8% | 8% | 9% | 6% | 7% |
| | Neutral | 22% | 25% | 23% | 16% | 17% | 21% | 33% | 23% |
| | Most effective | 68% | 62% | 65% | 76% | 75% | 70% | 61% | 71% |
| Round Table Meeting/focused group meetings | Least effective | 12% | 9% | 3% | 2% | 10% | 10% | 9% | 7% |
| | Neutral | 24% | 26% | 22% | 26% | 21% | 24% | 18% | 14% |
| | Most effective | 64% | 65% | 76% | 72% | 69% | 67% | 72% | 79% |
| Conferences | Least effective | 9% | 7% | 3% | 4% | 13% | 7% | 12% | 7% |
| | Neutral | 18% | 20% | 23% | 20% | 28% | 28% | 31% | 11% |
| | Most effective | 72% | 72% | 73% | 76% | 59% | 66% | 56% | 81% |
| Case Presentations | Least effective | 5% | 4% | 5% | 6% | 6% | 6% | 8% | 12% |
| | Neutral | 23% | 31% | 18% | 14% | 18% | 19% | 28% | 19% |
| | Most effective | 72% | 65% | 77% | 80% | 75% | 75% | 62% | 70% |

## Slide 3
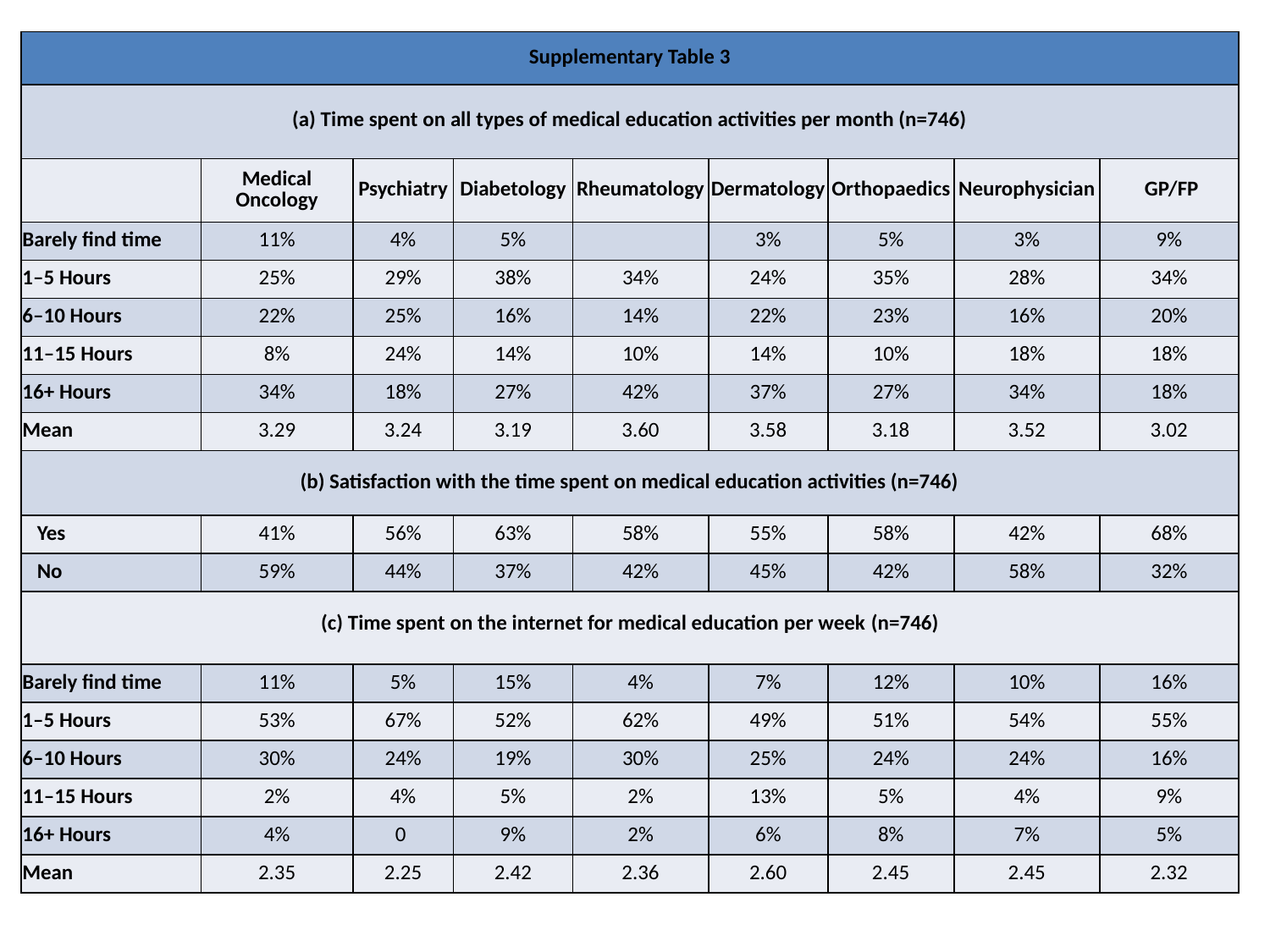

| Supplementary Table 3 | | | | | | | | |
| --- | --- | --- | --- | --- | --- | --- | --- | --- |
| (a) Time spent on all types of medical education activities per month (n=746) | | | | | | | | |
| | Medical Oncology | Psychiatry | Diabetology | Rheumatology | Dermatology | Orthopaedics | Neurophysician | GP/FP |
| Barely find time | 11% | 4% | 5% | | 3% | 5% | 3% | 9% |
| 1–5 Hours | 25% | 29% | 38% | 34% | 24% | 35% | 28% | 34% |
| 6–10 Hours | 22% | 25% | 16% | 14% | 22% | 23% | 16% | 20% |
| 11–15 Hours | 8% | 24% | 14% | 10% | 14% | 10% | 18% | 18% |
| 16+ Hours | 34% | 18% | 27% | 42% | 37% | 27% | 34% | 18% |
| Mean | 3.29 | 3.24 | 3.19 | 3.60 | 3.58 | 3.18 | 3.52 | 3.02 |
| (b) Satisfaction with the time spent on medical education activities (n=746) | | | | | | | | |
| Yes | 41% | 56% | 63% | 58% | 55% | 58% | 42% | 68% |
| No | 59% | 44% | 37% | 42% | 45% | 42% | 58% | 32% |
| (c) Time spent on the internet for medical education per week (n=746) | | | | | | | | |
| Barely find time | 11% | 5% | 15% | 4% | 7% | 12% | 10% | 16% |
| 1–5 Hours | 53% | 67% | 52% | 62% | 49% | 51% | 54% | 55% |
| 6–10 Hours | 30% | 24% | 19% | 30% | 25% | 24% | 24% | 16% |
| 11–15 Hours | 2% | 4% | 5% | 2% | 13% | 5% | 4% | 9% |
| 16+ Hours | 4% | 0 | 9% | 2% | 6% | 8% | 7% | 5% |
| Mean | 2.35 | 2.25 | 2.42 | 2.36 | 2.60 | 2.45 | 2.45 | 2.32 |

## Slide 4
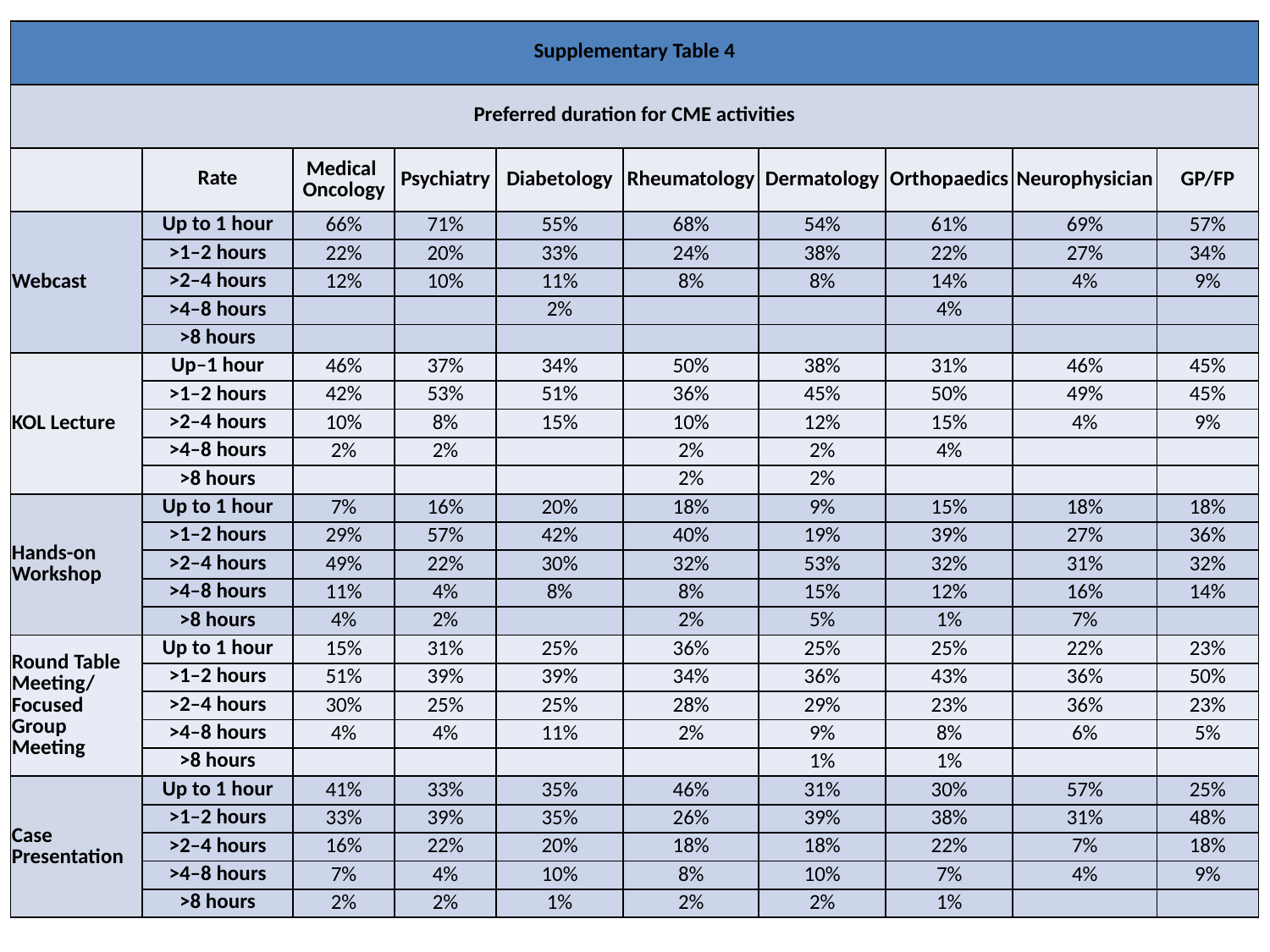

| Supplementary Table 4 | | | | | | | | | |
| --- | --- | --- | --- | --- | --- | --- | --- | --- | --- |
| Preferred duration for CME activities | | | | | | | | | |
| | Rate | Medical Oncology | Psychiatry | Diabetology | Rheumatology | Dermatology | Orthopaedics | Neurophysician | GP/FP |
| Webcast | Up to 1 hour | 66% | 71% | 55% | 68% | 54% | 61% | 69% | 57% |
| | >1–2 hours | 22% | 20% | 33% | 24% | 38% | 22% | 27% | 34% |
| | >2–4 hours | 12% | 10% | 11% | 8% | 8% | 14% | 4% | 9% |
| | >4–8 hours | | | 2% | | | 4% | | |
| | >8 hours | | | | | | | | |
| KOL Lecture | Up–1 hour | 46% | 37% | 34% | 50% | 38% | 31% | 46% | 45% |
| | >1–2 hours | 42% | 53% | 51% | 36% | 45% | 50% | 49% | 45% |
| | >2–4 hours | 10% | 8% | 15% | 10% | 12% | 15% | 4% | 9% |
| | >4–8 hours | 2% | 2% | | 2% | 2% | 4% | | |
| | >8 hours | | | | 2% | 2% | | | |
| Hands-on Workshop | Up to 1 hour | 7% | 16% | 20% | 18% | 9% | 15% | 18% | 18% |
| | >1–2 hours | 29% | 57% | 42% | 40% | 19% | 39% | 27% | 36% |
| | >2–4 hours | 49% | 22% | 30% | 32% | 53% | 32% | 31% | 32% |
| | >4–8 hours | 11% | 4% | 8% | 8% | 15% | 12% | 16% | 14% |
| | >8 hours | 4% | 2% | | 2% | 5% | 1% | 7% | |
| Round Table Meeting/ Focused Group Meeting | Up to 1 hour | 15% | 31% | 25% | 36% | 25% | 25% | 22% | 23% |
| | >1–2 hours | 51% | 39% | 39% | 34% | 36% | 43% | 36% | 50% |
| | >2–4 hours | 30% | 25% | 25% | 28% | 29% | 23% | 36% | 23% |
| | >4–8 hours | 4% | 4% | 11% | 2% | 9% | 8% | 6% | 5% |
| | >8 hours | | | | | 1% | 1% | | |
| Case Presentation | Up to 1 hour | 41% | 33% | 35% | 46% | 31% | 30% | 57% | 25% |
| | >1–2 hours | 33% | 39% | 35% | 26% | 39% | 38% | 31% | 48% |
| | >2–4 hours | 16% | 22% | 20% | 18% | 18% | 22% | 7% | 18% |
| | >4–8 hours | 7% | 4% | 10% | 8% | 10% | 7% | 4% | 9% |
| | >8 hours | 2% | 2% | 1% | 2% | 2% | 1% | | |

## Slide 5
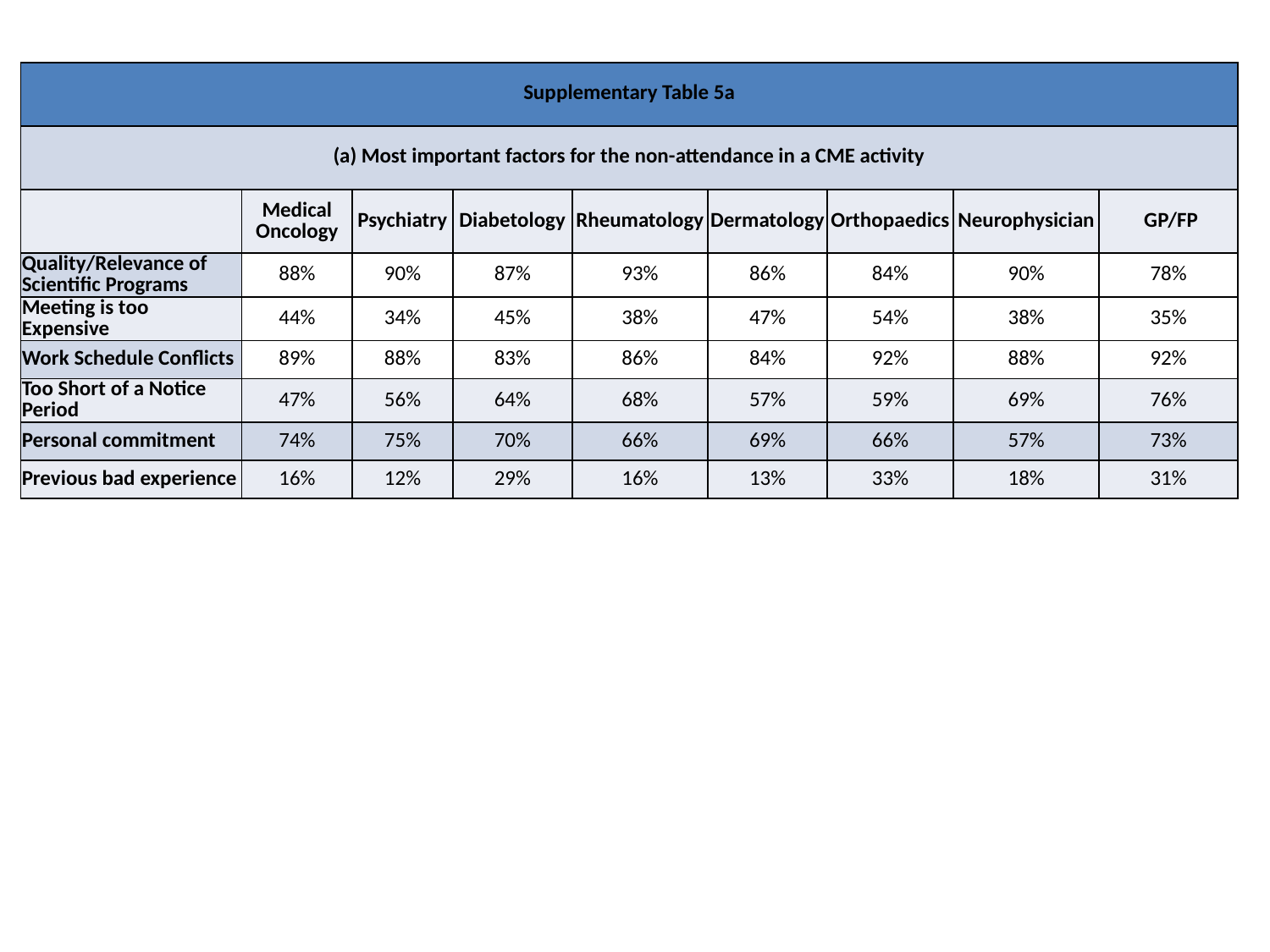

| Supplementary Table 5a | | | | | | | | |
| --- | --- | --- | --- | --- | --- | --- | --- | --- |
| (a) Most important factors for the non-attendance in a CME activity | | | | | | | | |
| | Medical Oncology | Psychiatry | Diabetology | Rheumatology | Dermatology | Orthopaedics | Neurophysician | GP/FP |
| Quality/Relevance of Scientific Programs | 88% | 90% | 87% | 93% | 86% | 84% | 90% | 78% |
| Meeting is too Expensive | 44% | 34% | 45% | 38% | 47% | 54% | 38% | 35% |
| Work Schedule Conflicts | 89% | 88% | 83% | 86% | 84% | 92% | 88% | 92% |
| Too Short of a Notice Period | 47% | 56% | 64% | 68% | 57% | 59% | 69% | 76% |
| Personal commitment | 74% | 75% | 70% | 66% | 69% | 66% | 57% | 73% |
| Previous bad experience | 16% | 12% | 29% | 16% | 13% | 33% | 18% | 31% |

## Slide 6
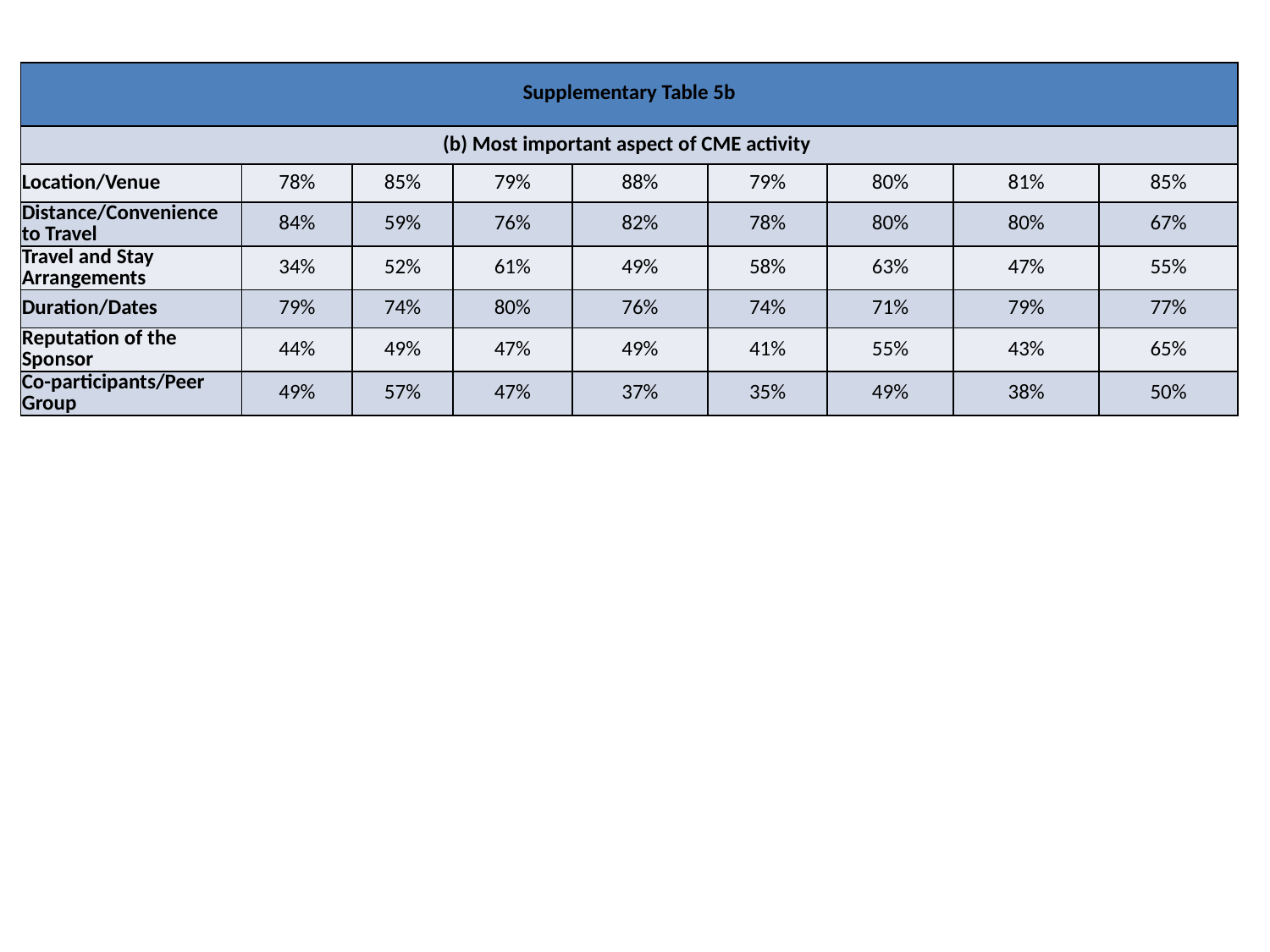

| Supplementary Table 5b | | | | | | | | |
| --- | --- | --- | --- | --- | --- | --- | --- | --- |
| (b) Most important aspect of CME activity | | | | | | | | |
| Location/Venue | 78% | 85% | 79% | 88% | 79% | 80% | 81% | 85% |
| Distance/Convenience to Travel | 84% | 59% | 76% | 82% | 78% | 80% | 80% | 67% |
| Travel and Stay Arrangements | 34% | 52% | 61% | 49% | 58% | 63% | 47% | 55% |
| Duration/Dates | 79% | 74% | 80% | 76% | 74% | 71% | 79% | 77% |
| Reputation of the Sponsor | 44% | 49% | 47% | 49% | 41% | 55% | 43% | 65% |
| Co-participants/Peer Group | 49% | 57% | 47% | 37% | 35% | 49% | 38% | 50% |

## Slide 7
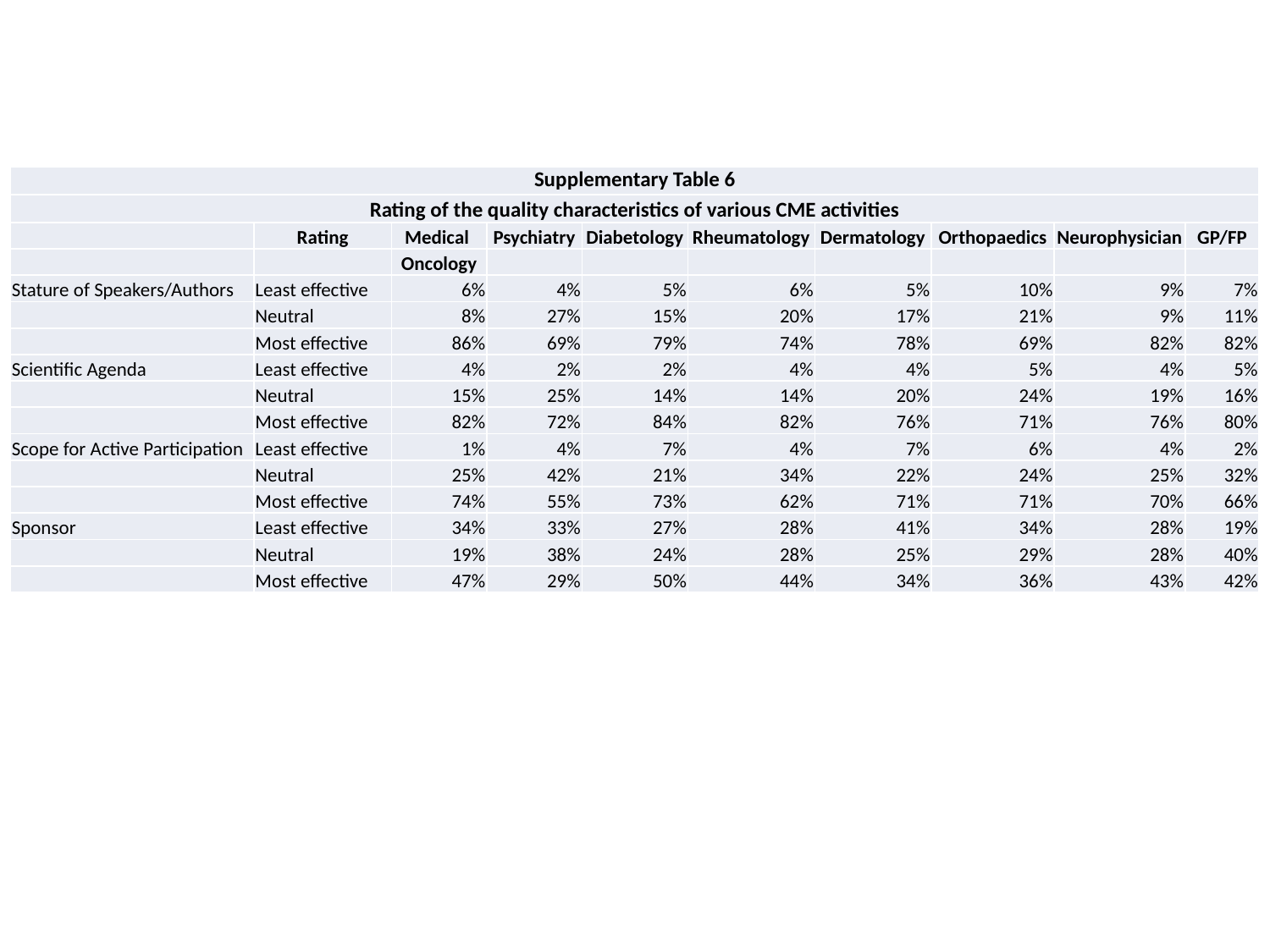

| Supplementary Table 6 | | | | | | | | | |
| --- | --- | --- | --- | --- | --- | --- | --- | --- | --- |
| Rating of the quality characteristics of various CME activities | | | | | | | | | |
| | Rating | Medical | Psychiatry | Diabetology | Rheumatology | Dermatology | Orthopaedics | Neurophysician | GP/FP |
| | | Oncology | | | | | | | |
| Stature of Speakers/Authors | Least effective | 6% | 4% | 5% | 6% | 5% | 10% | 9% | 7% |
| | Neutral | 8% | 27% | 15% | 20% | 17% | 21% | 9% | 11% |
| | Most effective | 86% | 69% | 79% | 74% | 78% | 69% | 82% | 82% |
| Scientific Agenda | Least effective | 4% | 2% | 2% | 4% | 4% | 5% | 4% | 5% |
| | Neutral | 15% | 25% | 14% | 14% | 20% | 24% | 19% | 16% |
| | Most effective | 82% | 72% | 84% | 82% | 76% | 71% | 76% | 80% |
| Scope for Active Participation | Least effective | 1% | 4% | 7% | 4% | 7% | 6% | 4% | 2% |
| | Neutral | 25% | 42% | 21% | 34% | 22% | 24% | 25% | 32% |
| | Most effective | 74% | 55% | 73% | 62% | 71% | 71% | 70% | 66% |
| Sponsor | Least effective | 34% | 33% | 27% | 28% | 41% | 34% | 28% | 19% |
| | Neutral | 19% | 38% | 24% | 28% | 25% | 29% | 28% | 40% |
| | Most effective | 47% | 29% | 50% | 44% | 34% | 36% | 43% | 42% |

## Slide 8
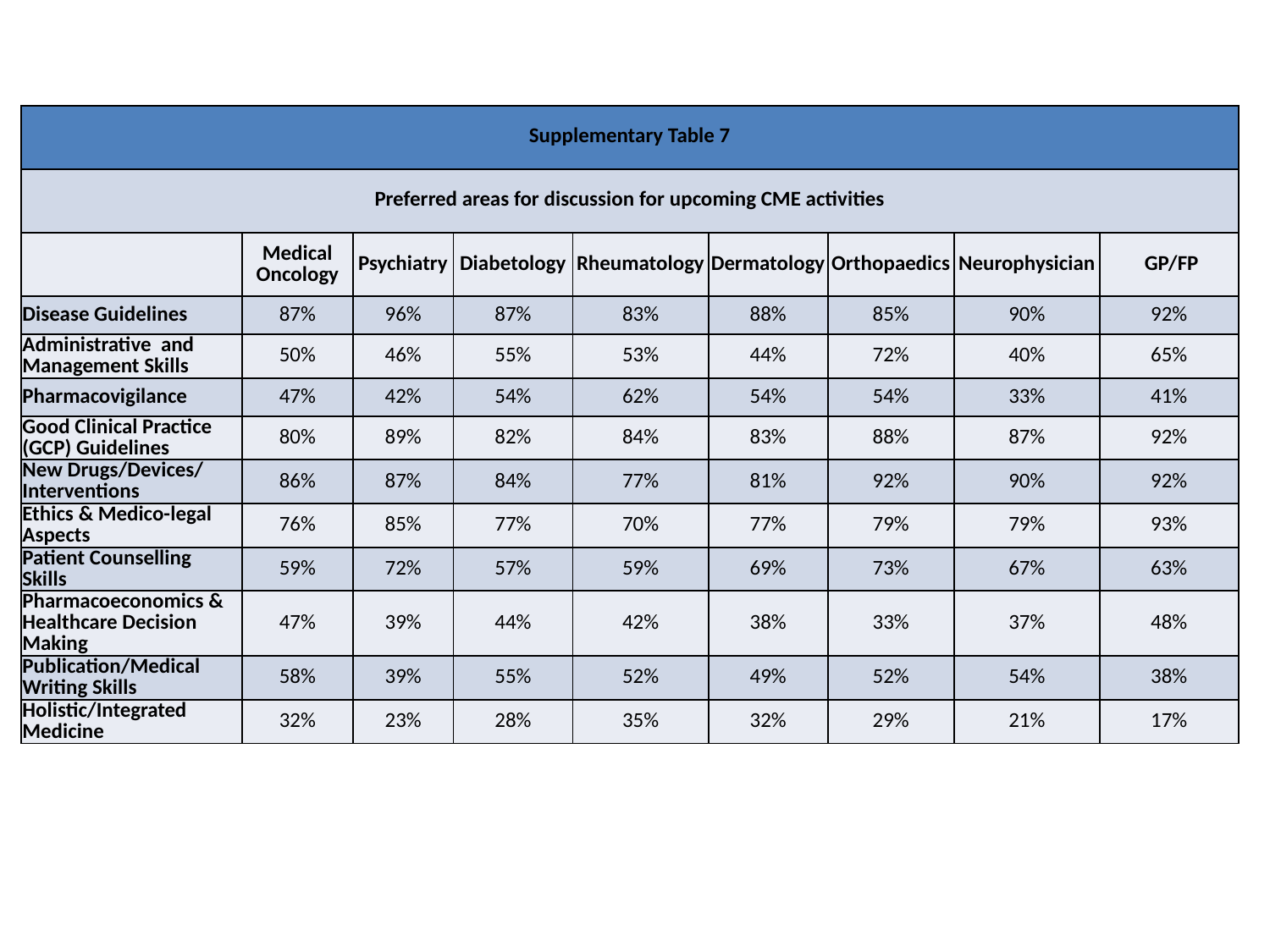

| Supplementary Table 7 | | | | | | | | |
| --- | --- | --- | --- | --- | --- | --- | --- | --- |
| Preferred areas for discussion for upcoming CME activities | | | | | | | | |
| | Medical Oncology | Psychiatry | Diabetology | Rheumatology | Dermatology | Orthopaedics | Neurophysician | GP/FP |
| Disease Guidelines | 87% | 96% | 87% | 83% | 88% | 85% | 90% | 92% |
| Administrative and Management Skills | 50% | 46% | 55% | 53% | 44% | 72% | 40% | 65% |
| Pharmacovigilance | 47% | 42% | 54% | 62% | 54% | 54% | 33% | 41% |
| Good Clinical Practice (GCP) Guidelines | 80% | 89% | 82% | 84% | 83% | 88% | 87% | 92% |
| New Drugs/Devices/ Interventions | 86% | 87% | 84% | 77% | 81% | 92% | 90% | 92% |
| Ethics & Medico-legal Aspects | 76% | 85% | 77% | 70% | 77% | 79% | 79% | 93% |
| Patient Counselling Skills | 59% | 72% | 57% | 59% | 69% | 73% | 67% | 63% |
| Pharmacoeconomics & Healthcare Decision Making | 47% | 39% | 44% | 42% | 38% | 33% | 37% | 48% |
| Publication/Medical Writing Skills | 58% | 39% | 55% | 52% | 49% | 52% | 54% | 38% |
| Holistic/Integrated Medicine | 32% | 23% | 28% | 35% | 32% | 29% | 21% | 17% |

## Slide 9
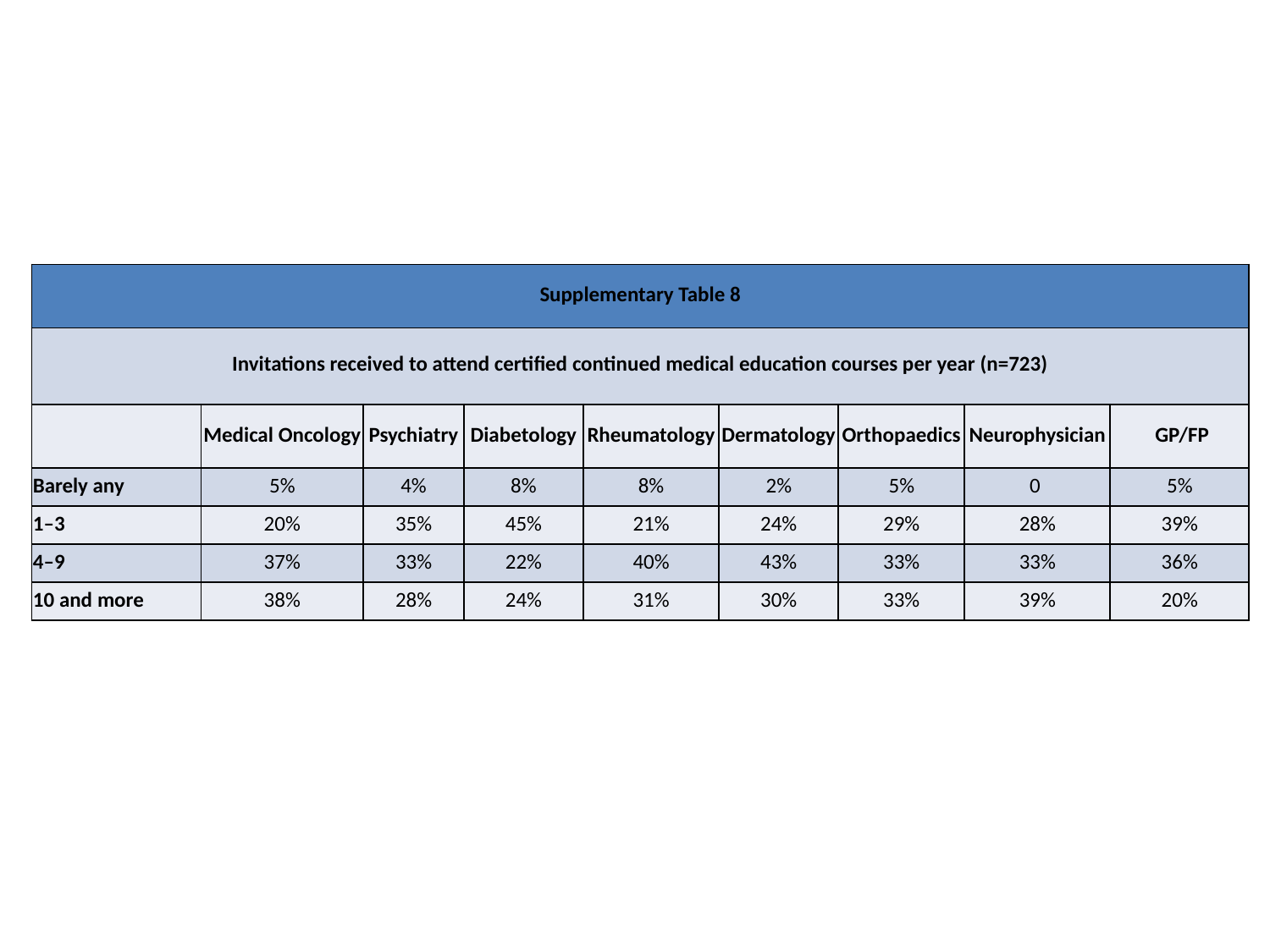

| Supplementary Table 8 | | | | | | | | |
| --- | --- | --- | --- | --- | --- | --- | --- | --- |
| Invitations received to attend certified continued medical education courses per year (n=723) | | | | | | | | |
| | Medical Oncology | Psychiatry | Diabetology | Rheumatology | Dermatology | Orthopaedics | Neurophysician | GP/FP |
| Barely any | 5% | 4% | 8% | 8% | 2% | 5% | 0 | 5% |
| 1–3 | 20% | 35% | 45% | 21% | 24% | 29% | 28% | 39% |
| 4–9 | 37% | 33% | 22% | 40% | 43% | 33% | 33% | 36% |
| 10 and more | 38% | 28% | 24% | 31% | 30% | 33% | 39% | 20% |
